# Various stress stimuli rewire the profile of liver secretome in a p53-dependent manner

**DOI:** 10.1038/s41419-018-0697-4

**Published:** 2018-05-29

**Authors:** Meital Charni-Natan, Hilla Solomon, Alina Molchadsky, Adi Jacob-Berger, Naomi Goldfinger, Varda Rotter

**Affiliations:** Department of Molecular Cell Biology, Weizmann Institute of Science, Rehovot, Israel

## Abstract

Liver is an important secretory organ that consistently manages various insults in order to retain whole-body homeostasis. Importantly, it was suggested that the tumor-suppressor p53 plays a role in a variety of liver physiological processes and thus it is being regarded as a systemic homeostasis regulator. Using high-throughput mass spectrometric analysis, we identified various p53-dependent liver secretome profiles. This allowed a global view on the role of p53 in maintaining the harmony of liver and whole-body homeostasis. We found that p53 altered the liver secretome differently under various conditions. Under physiological conditions, p53 controls factors that are related mainly to lipid metabolism and injury response. Upon exposure to various types of cancer therapy agents, the hepatic p53 is activated and induces the secretion of proteins related to additional pathways, such as hemostasis, immune response, and cell adhesion. Interestingly, we identified a possible relationship between p53-dependent liver functions and lung tumors. The latter modify differently liver secretome profile toward the secretion of proteins mainly related to cell migration and immune response. The notion that p53 may rewire the liver secretome profile suggests a new non-cell autonomous role of p53 that affect different liver functions and whole organism homeostasis.

## Introduction

It is well accepted that the tumor-suppressor p53 is activated upon various stress stimuli^1^. Depending on the stress source and amplitude, p53 activates various molecular pathways^1,2^. p53 canonical processes include cell cycle arrest, apoptosis, and senescence. However, recent accumulating data demonstrate that p53 exerts additional important non-canonical functions mainly associated with the cell surrounding such as regulating the tumor microenvironment, metastasis, and metabolism^1,3^. Furthermore, it was suggested that expression of p53 in the liver controls the entire organism homeostasis^4–6^. Notably, the liver is a central metabolic organ, which performs a plethora of metabolic functions, such as glycogen storage, decay of red blood cells, and synthesis and secretion of many factors including vitamins and hormones. The physiological role of the liver entails the regulation of plasma component homeostasis and the elimination of toxic metabolites such as drugs that can be destructive to the tissue and eventually to the entire body^7–9^. Thus the fact that p53 was found to regulate many processes in the liver including drugs, glucose and lipids metabolism may suggest p53 as a regulator of systemic homeostasis^4,10–12^. Furthermore, the liver serves as a major secretory gland^7^. Approximately 4% of all human protein coding genes are specifically expressed in the liver, where 33% of them are secreted to the plasma, and are related to hemostasis and fibrinolysis, carrier proteins, and enzymes^13,14^. Among the secreted factors are protein related to senescence-associated secretory phenotype (SASP) found to be induced by hepatic p53 and to affect the surrounding liver tissue. This non-cell autonomous activity of p53 may attenuate liver fibrosis and liver tumor progression^15–18^. Recently, it was demonstrated that in response to distal lung tumor, the liver exhibited changes in its secretome, which affect the whole-body homeostasis^19^. Interestingly, in our previous work we reported a reciprocal liver–tumor connection. We observed that activated hepatic p53 induced the secretion of sex hormone-binding globulin (SHBG), which can attenuate breast cancer cells' survival^5^. In all, these observations suggest an important role for p53 as a regulator of the entire organism homeostasis by mediating the secretion of key factors of the liver. Despite the extensive effort to decipher the numerous outcomes of the activated hepatic p53, its involvement in liver secretome has not yet been clarified. In the present study, we utilized high-throughput mass spectrometric (MS) analysis on hepatic cell line media, which led us to uncover various liver secretome profiles governed by p53. While physiological activity of the hepatic p53 resulted in the secretion of factors that participate in normal liver functions, exposure to drugs and chemotherapies activate the hepatic p53, which in turn altered the secretion profile of the liver. p53 activation induced the secretion of proteins related to insulin, glucocorticoids, and extracellular matrix (ECM) modulators with a focus on cell adhesion and regulation of immune response. In addition, our in vivo study demonstrated that the presence of lung tumors correlated with hepatic p53 activation and liver malfunctioning. Our corresponding in vitro model for liver–tumor interaction identified an additional p53-dependent secretion profile. These secreted factors are mainly related to immune response and cell migration, implying an interesting relation between a distal tumor and the liver. Data derived from this study unravel an important angle of p53 both under physiological and pathological conditions, as a systemic regulator of the global organism homeostasis and on its non-cell autonomous affects in the liver.

## Results

### Hepatic p53 regulates the level of secreted proteins related to liver physiology

**Fig. 1:**
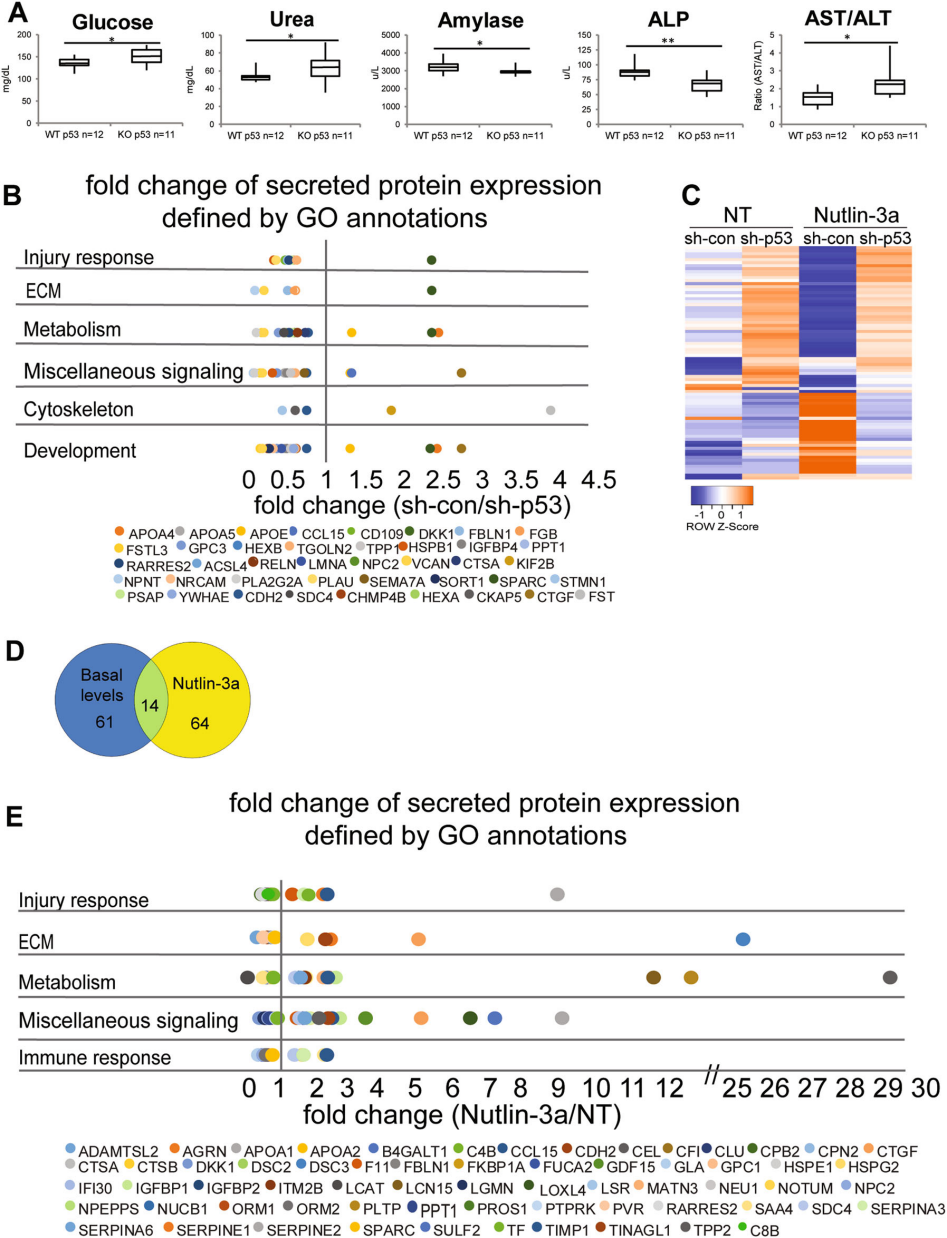
p53 activation alters the profile of the p53-dependent liver secreted proteins. **a** Analysis glucose, urea, amylase ALP, and AST/ALT concentrations in sera obtained from WTp53 and p53 KO mice are represented in box plot. Average (horizontal line), standard deviation (box), and highest and lowest reads (error bars) are shown. (*t*-test, *P < 0.05, **P < 0.01, WTp53 mice *n* = 12, p53 KO mice *n* = 11). **b** MS analysis was performed on CM from HepG2 sh-con/sh-p53 cells. The secreted proteins that were significantly dependent on p53 [(sh-con/sh-p53) <0.75 or >1.3, *t*-test *P* < 0.05, *n* = 3 independent experiments] were furthered analyzed for their GO biological annotations by gene-card analysis tool (GeneAnalytics). Different GO annotations were grouped to major categories as presented in the graph (fold change are listed in Table S1, annotations are listed in Table S2). Each dot represents different secreted protein, distributed by its relevant fold change (sh-con/sh-p53) (downregulated proteins <1, upregulated proteins >1). **c**–**e** MS analysis was performed on CM from HepG2 sh-con/sh-p53 cells that were treated with 10 µM Nutlin-3a. The secreted proteins that were further analyzed are the ones whose levels were significantly altered in p53-dependent manner upon Nutlin-3a treatment. First, we filtered for the proteins whose expression was altered upon Nutlin-3a treatment in sh-con cells [(sh-con treated/sh-con non-treated) <0.75 or >1.3, *t*-test *P* < 0.05] and then we included only the proteins that were significantly induced in p53-expressing cells ((sh-con treated/sh-p53 treated) or (fold change sh-con/fold change sh-p53) *t*-test *P* < 0.05. *n* = 3 independent experiments). **c** Heatmap showing hierarchical clustering of these secreted proteins. Each row represents different protein and columns represent different treatment or cells’ type. Proteins' intensities (protein abundances) were clustered using a Pearson method. Colors reflect *z*-value standard deviations (−1 to +1). The secreted proteins and their relevant fold change are listed Table S4. **d** The secreted proteins that significantly changed by p53 physiological conditions (Table S1) and by Nutlin-3a treatment (Table S4) are represented in Venn-diagram. **e** The secreted proteins that were significantly dependent on p53 were affiliated by gene-card analysis tool (GeneAnalytics) to their GO biological annotations. Different GO annotations were grouped to major categories as presented in the graph (annotations are listed in Table S5). Each dot represents different secreted protein, distributed by its relevant fold change (sh-con/sh-p53) (downregulated proteins <1, upregulated proteins >1)

Our previous study showed that p53 participates in homeostasis maintenance by regulating proteins secretion to mice sera^5^. In an attempt to better understand this role of p53, we compared various blood biochemical parameters obtained from wild-type p53 (WTp53) and p53 knockout (p53 KO) mice sera^20^. We showed significant variations in the levels of glucose, urea, amylase, Alkaline phosphatase (ALP), alanine aminotransferase (ALT), and aspartate aminotransferase (AST) (Fig. 1a), suggesting that p53 can affect global homeostasis. Interestingly, alterations in the serum concentrations and in the ratio of ALP, ALT, and AST commonly indicate liver malfunction^21^. These findings imply an important role of p53 in regulating liver normal homeostasis. Hence, to obtain a more global view on the mechanism underlying hepatic p53 function, we focused on studying p53-dependent liver secretion profiles. To that end, we adopted a proteomic high-throughput approach, based on MS. To identify the hepatic p53-dependent secreted proteins, we used an in vitro model of human liver cells, HepG2, in which p53 was knocked down by short hairpin RNA (shRNA) (sh-p53) or left intact (sh-con) (Figure S1). Conditioned media (CM) of the various cell lines were collected and subjected to MS analysis. Out of the total 1451 secreted proteins identified, 75 were significantly p53 dependent. Out of these, 12 showed a higher than 1.3 sh-con/sh-p53 fold change (i.e., upregulated) while 63 showed a lower than 0.75 sh-con/sh-p53 fold change (i.e., downregulated) (Table S1). To affiliate the secreted proteins by their biological annotation, we used the gene-card analysis tool, GeneAnalytics^22^. This analysis revealed that most of the biological pathways are related to normal liver applications of homeostasis maintenance such as lipids metabolism and response to injury (Fig. 1b, Table S2). Indeed, liver disease can affect the blood flow and cause significant bleeding problems^23^. Interestingly, we found that most of the secreted proteins are downregulated by p53 (Fig. 1b). Taking together, both the in vivo and in vitro experiments suggest that under physiological conditions p53 regulates the expression of liver-secreted factors that are necessary for normal liver functions and homeostasis maintenance.

### p53 activation alters the profile of the p53-dependent liver-secreted proteins

**Fig. 2:**
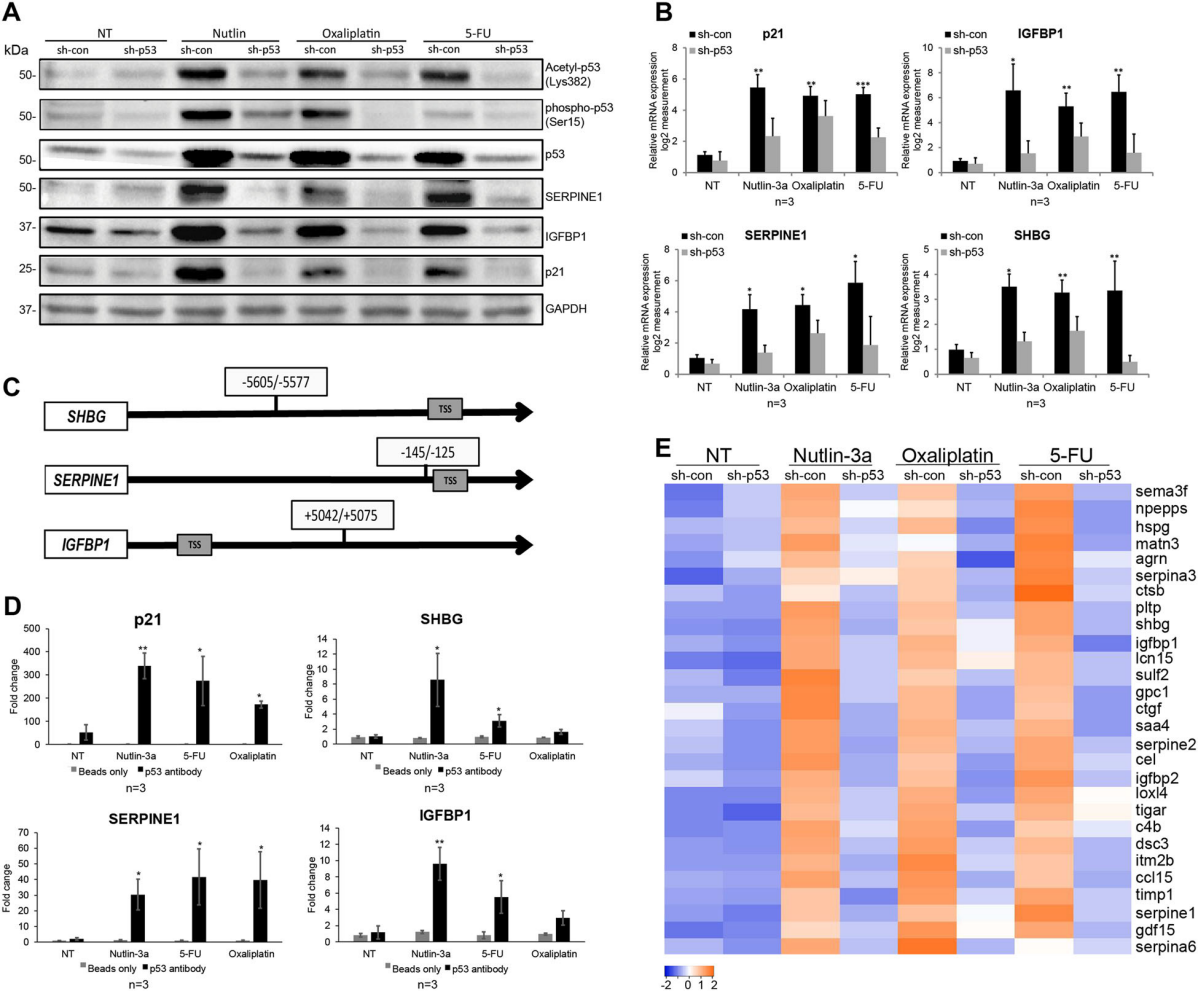
Different chemotherapy treatments affect the hepatic p53 activation and its secretion profile. HepG2 sh-con/sh-p53 cells were either treated with 10 µM Nutlin-3a, 5 µM Oxaliplatin, and 50 µM Fluorouracil (5-FU) for 24 h or supplemented with DMSO and non-treated (NT) as a control. **a** Protein levels of Accetyl-p53 (Lys382), phosphor-p53 (Ser15), p53, SERPINE1, IGFBP1, and p21 were measured by western blot. GAPDH was used as a loading control. The blot is representative of at least three experiments. **b** mRNA levels of *p21*, *IGFBP1*, *SERPINE1*, and *SHBG* were measured by qRT-PCR analysis. Results presented as mean ± SE (*t*-test, *P < 0.05, **P < 0.01, ***P < 0.001, *n* = 3 independent experiments). **c** SERPINE1 and IGFBP1 genomic sequences were analyzed by p53MH algorithm. Representative scheme of the potential p53 RE locations relative to TSS are indicated. The previously suggested RE in SHBG promoter^5^ served as a positive control. **d** ChIP analysis of HepG2 cells. p53 protein was immunoprecipitated using p53-specific H47 polyclonal antibody (p53 antibody) or with beads only as a control. qRT-PCR was performed using specific primers against the p53 REs indicated in **c**. Values were normalized to 1% input of the corresponding sample. Results presented as mean ± SE (*t*-test, *P < 0.05, **P < 0.01, *n* = 3 independent experiments). **e** Heatmap showing hierarchical clustering of the mRNA levels (measured by qRT-PCR analysis) of the genes that their proteins levels were significantly induced in a p53-dependent manner upon Nutlin-3a treatment. Each row represents different genes and columns represent different treatments or cells’ type. Genes mRNA levels were clustered using a Pearson method. Colors reflect *z*-value standard deviations (−2 to +2). Results were calculated as mean of three independent experiments

Next, we examine whether stress-activated p53 would affect liver secretion profile. To this end, we treated the HepG2 cells with 10 µM Nutlin-3a, a compound that stabilizes p53 protein^24^. As expected, a marked accumulation of p53 protein was detected in HepG2 sh-con compared to sh-p53 (Fig. 2a). To further assess the repertoire of secreted factors, we preformed MS analysis on CM-obtained HepG2 upon Nutlin-3a treatment. This analysis revealed 78 p53-dependent secreted proteins. Notably, p53 activation led to an increased number of upregulated proteins than that observed under p53 physiological conditions (34 vs. 12, respectively; Fig. 1c, Table S4). Moreover, while approximately 82% out of the secreted proteins were specific for Nutlin-3a treatment, only 18% of them were shared with those induced by p53 physiological conditions (Fig. 1d). Indeed, biological annotation analysis indicated that they are involved in additional physiological processes beyond those identified under non-activated p53 conditions. For example, we detected augmented representation of liver metabolic functions and pathways related to immune response (Fig. 1e, Table S5). This suggests that p53 regulates different secreted protein profiles when it is activated, compared with its mediated secretion under physiological conditions.

### Different chemotherapy treatments affect the hepatic p53 activation and its secretion profile

The liver regulates metabolism of drugs and chemicals subjected to the body^8^. Consequently, the hepatic p53 may be activated, as we previously reported^5^, and induce the expression of enzymes responsible for systemic clearance^10^. To examine this, we treated the HepG2 cells with either Oxaliplatin (5 µM), Fluorouracil (5-FU; 50 µM), or with Nutlin-3a as a positive control for p53 activation. As expected, we detected p53 protein stabilization (Fig. 2a). Moreover, to assess p53 activation, we analyzed its posttranslational modifications, Serine 15 and Lysine 382, that indicates its activity^25–28^. Indeed, phosphorylated/acetylated p53 was elevated upon treatments. To validate p53 activity, we measured both protein and mRNA levels of the p53 bona fide target gene p21 (Fig. 2a, b)^29^. Moreover, we quantified the expression of two proteins that are known as hepatic p53-secreted targets: insulin-growth factor-binding protein 1 (IGFBP1)^30^ and Serpin Family-E Member (SERPINE1)^31,32^. Indeed, we detected increased expression levels of these proteins (Fig. 2a), suggesting that p53 is accumulated and activated upon various chemotherapies. As a transcription factor, we examined whether p53 induces transcription of its hepatic-secreted targets. Therefore, we measured the mRNA levels of IGFBP1, SERPINE1, and additional p53 known secreted target SHBG^5^. Higher induction of the mRNA levels of these genes following Nutlin-3a and chemotherapy treatments were observed. This indicates that p53 induces these genes' expression, which further led to their higher mRNA and secreted proteins levels (Fig. 2b). To decipher the mechanism by which p53 regulates these genes, we used the p53MH algorithm^33^ to screen for p53 responsive elements (REs) entailed in the chromatin of SEREPINE1 and IGFBP1. Indeed, p53 REs were detected, suggesting their potential as p53-direct targets (Fig. 2c). Moreover, to determine whether p53 is able to bind these REs, we performed chromatin immunoprecipitation (ChIP) following the above-mentioned treatments. Indeed, we found an enrichment for these p53 REs in all the examined secreted targets and in p21 (Fig. 2d). Altogether, these data suggest that p53 induces the expression of the secreted proteins by directly binding to their chromatin via specific REs.

Next, we examined whether the Nutlin-3a-dependent protein secretion profile of HepG2 cells is also expressed following chemotherapy treatments. We analyzed the mRNA levels of the upregulated secreted proteins detected by the MS analysis upon Nutlin-3a treatment (Fig. 1c). Strikingly, 28 out of the 34 genes showed a similar expression pattern as observed following Nutlin-3a (Fig. 2e). This implies that specific chemotherapy agents lead to similar induction effect. Overall, these data suggest that treatment with both Nutlin-3a and chemotherapies led to accumulation and activation of the hepatic p53 protein, which in turn affects the liver secretome by inducing several secreted proteins encoded by genes' expression.

### p53 is activated in the livers of mice bearing lung tumors

**Fig. 3:**
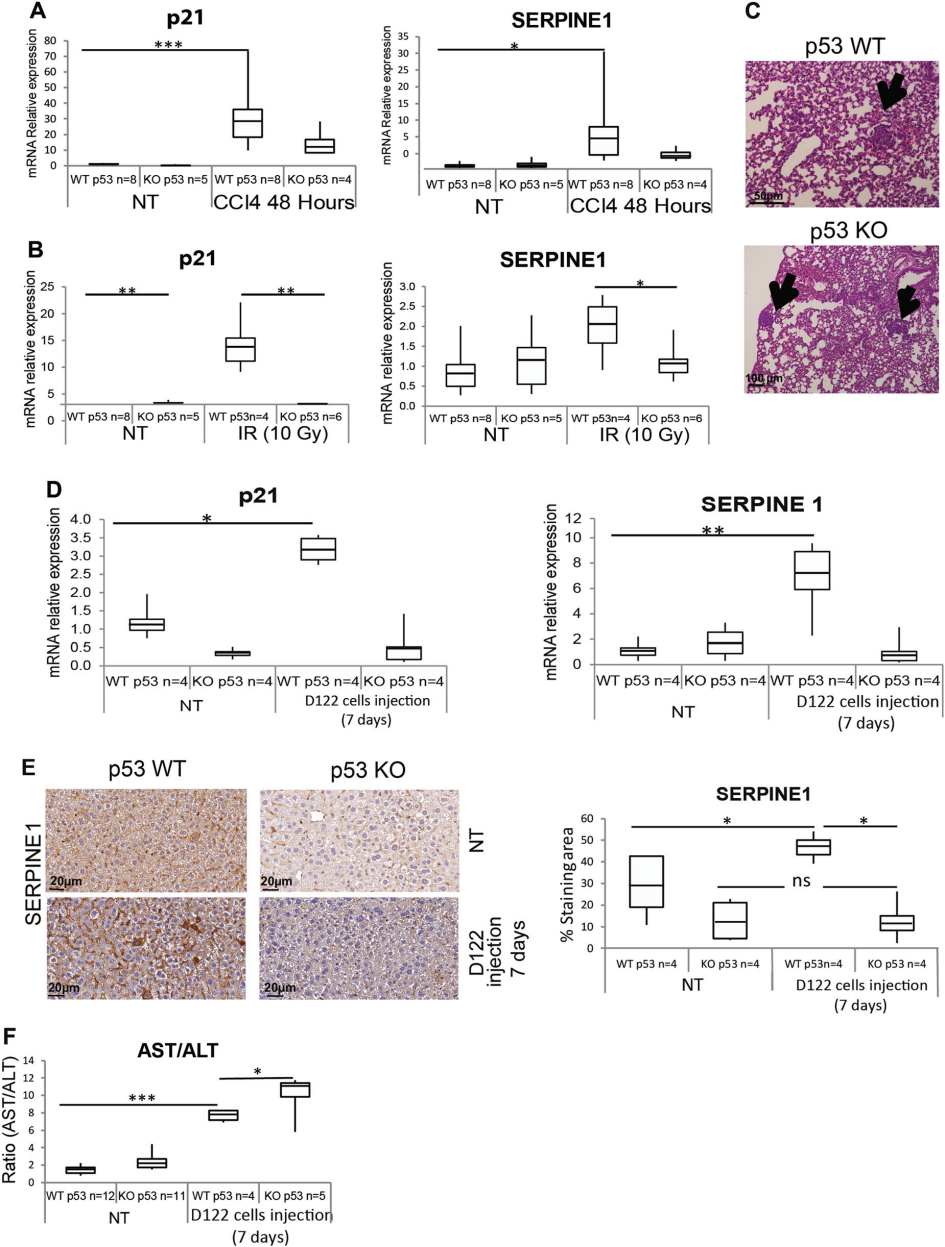
Mice bearing lung tumors exhibit elevated expression levels of p53 target genes in their livers. **a** WTp53 and p53 KO mice were intraperitoneally injected with CCl_4_ or with sterile corn oil as a control (NT). After 48 h, mice livers were extracted, total RNA was isolated from the livers, and mRNA levels of p21 and SERPINE1 were measured by qRT-PCR. The analyzed mRNA levels are represented in box plot. Average (horizontal line), standard deviation (box), and highest and lowest reads (error bars) are shown. Unpaired *t*-test, *P < 0.05, ***P < 0.001. WTp53 NT *n* = 8 mice, p53 KO NT *n* = 5 mice, WTp53 treated *n* = 8 mice, p53 KO treated *n* = 4 mice). **b** WTp53 and p53 KO mice were irradiated (IR, 10 Gy) or left non-treated as a control (NT). After 24 h, mice livers were extracted, total RNA was isolated from mice livers, and mRNA levels of p21 and SERPINE1 were measured by qRT-PCR. The analyzed mRNA levels are represented in box plot. Average (horizontal line), standard deviation (box), and highest and lowest reads (error bars) are shown. (Unpaired *t*-test, *P < 0.05, **P < 0.01. WTp53 NT *n* = 8 mice, p53 KO NT *n* = 5 mice, WTp53 treated *n* = 4 mice, p53 KO treated *n* = 6 mice). **c**–**f** WTp53 and p53 KO mice were intravenously injected with D122 cancer cells (10^6^ cells) or with PBS alone as a control (NT). After 7 days, lung tumors were generated and mice were sacrificed. *n* = 4 mice. **c** Representative lung histological sections stained by H&E of D122 cell-derived tumors. Arrows indicate a cluster of neoplastic cells in the mice lungs. **d** Mice livers were extracted and total RNA was isolated. mRNA levels of p21 and SERPINE1 were measured by qRT-PCR and are represented in box plot. Average (horizontal line), standard deviation (box), and highest and lowest reads (error bars) are shown. Unpaired *t*-test, *P < 0.05, **P < 0.01. WTp53 NT *n* = 4 mice, p53 KO NT *n* = 4 mice, WTp53 treated *n* = 4 mice, p53 KO treated *n* = 4 mice. **e** Liver sections were immunohistochemically stained with an anti-SERPINE1 antibody combined with a biotinylated secondary antibody (brown; DAB). Left panel shows representative stained liver sections (additional photos presented in Figure S4). Right panel presents quantification of SERPINE1 staining area in box plot. Average (horizontal line), standard deviation (box), and highest and lowest reads (error bars) are shown. Unpaired *t*-test, *P < 0.05. WTp53 NT *n* = 4 mice, p53 KO NT *n* = 4 mice, WTp53 treated *n* = 4 mice, p53 KO treated *n* = 4 mice). **f** Mice sera were collected and the concentrations of AST and ALT enzymes were analyzed. AST/ALT ratio of WTp53 and p53 KO mice are represented in box plot. Average (horizontal line), standard deviation (box), and highest and lowest reads (error bars) are shown. Unpaired *t*-test, *P < 0.05, ***P < 0.01. WTp53 NT *n* = 12 mice, p53 KO NT *n* = 11 mice, WTp53 treated *n* = 4 mice, p53KO treated *n* = 5 mice

To examine whether hepatic p53 can be activated and induce the expression of secreted proteins in physiological in vivo model, we compared WTp53 and p53 knockout (KO) transgenic mice. To this end, we subjected these mice to acute damage. To gain specific liver damage, mice were subjected to carbon tetrachloride (CCl_4_)^34^, whereas in order to gain general damage, mice were subjected to irradiation (IR). Forty eight or 24 h after CCl_4_ or IR treatments (respectively), mRNA levels of SERPINE1 and p21 in the mice livers were analyzed. Augmented levels of the genes were observed in WTp53 mice as compared with p53 KO mice (Fig. 3a, b). These suggest that, upon liver-specific damage or whole-body IR, hepatic p53 is activated and induces the expression of its targets.

A possible crosstalk between the liver and lung tumors was suggested^19,35^. Thus, to examine whether p53 might be involved, we studied whether lung tumors would activate the hepatic p53, which may affect the liver secretome. To this end, we induced lung tumors in WTp53 and p53 KO mice by intravenous injection of D122 cells, which have the capacity to form lung metastasis^36^. Seven days later, mice developed lung tumors (Fig. 3c) with no detectable neoplastic cells in their livers (Figure S2). Then mice were sacrificed and their livers were analyzed for p53 target gene expression, testifying for p53 activation. Indeed, significantly elevated levels of p21 and SERPINE1 mRNA were observed in the livers of WTp53 compared with p53 KO mice (Fig. 3d and Figure S3). Notably, WTp53 mice livers exhibited a more intensive immuno-staining of SERPINE1 protein both in liver cells and in the ECM, compared with p53 KO (Fig. 3e and Figure S4). These results suggest that hepatic p53 is activated in response to distal lung tumors and further promotes the expression of its cellular and secreted targets. To understand how p53 activity affects liver function in response to lung tumors' presence, we analyzed mice sera for liver-secreted enzymes. An augmented level of the ratio of AST/ALT was observed in both WTp53 and p53 KO mice bearing lung tumors compared to non-treated mice, testifying liver damage. Interestingly, the group of p53 KO presented a significant higher level of AST/ALT ratio as compared to WTp53 mice (Fig. 3f). This suggests that p53 may attenuate the liver dysfunction following the presence of distal tumors in the lungs.

### Human lung tumor cells induces hepatic p53 activation and alterations in the p53-dependent protein secretion profile

**Fig. 4:**
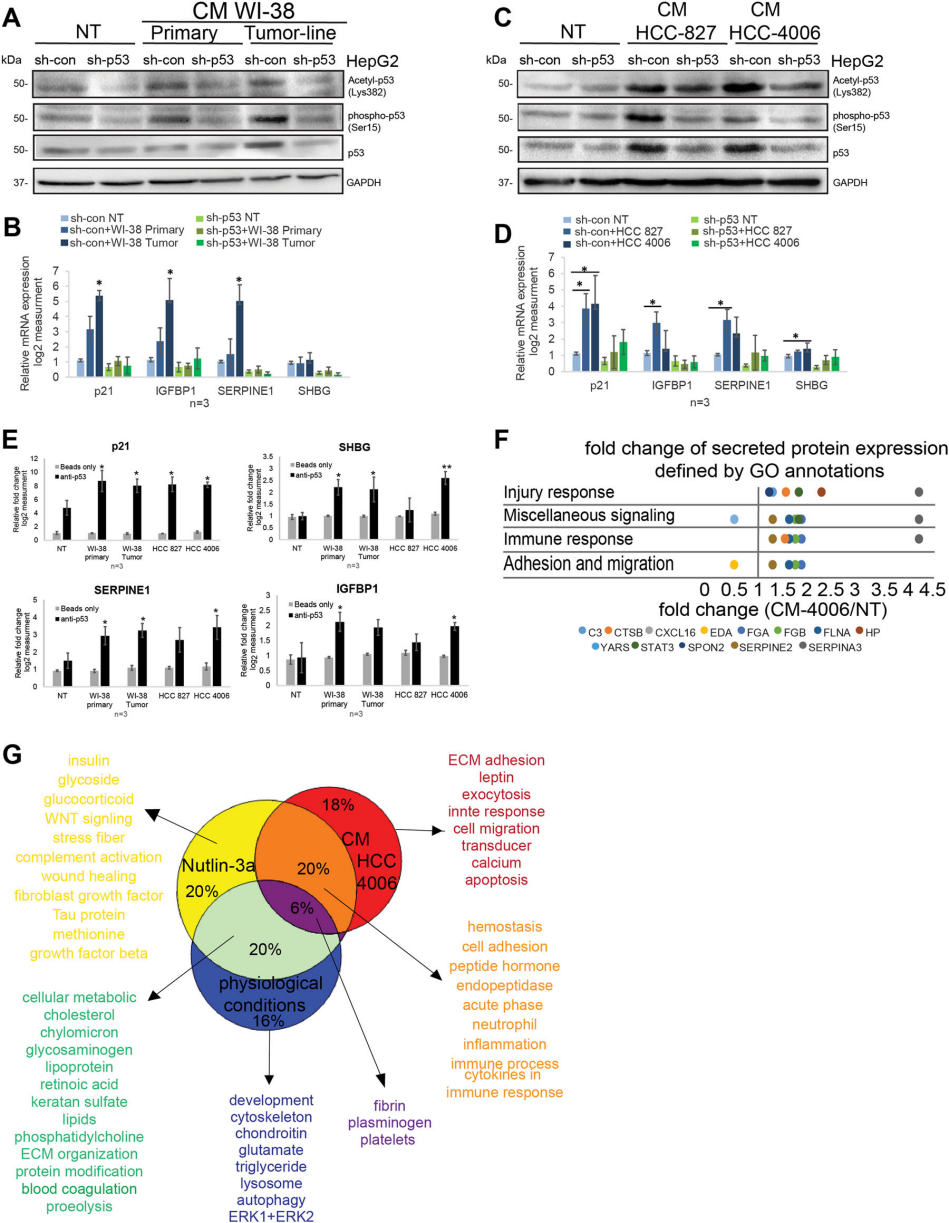
Conditioned media derived from human lung cancer cells induce hepatic p53 activation that leads to alterations in the protein secretion profile. HepG2 sh-con/sh-p53 cells were collected 24 h after treatments with either CM of primary WI-38 cells and WI-38 tumor-derived cell lines (**a**) or CM collected from lung cancer-derived cell lines, HCC 827 and HCC 4006, or left non-treated (NT) (**b**). **a**, **c** Protein levels of Accetyl-p53 (Lys382), phosphor-p53 (Ser15), and p53 were measured by western blot. GAPDH were used as a loading control. The blot is representative of at least three independent experiments. **b**, **d** mRNA levels of *p21*, *IGFBP1*, *SERPINE1*, and *SHBG* were measured by qRT-PCR analysis. Results presented as mean ± SE. *t*-test, *P < 0.05, **P < 0.01. *n* = 3 independent experiments. **e** ChIP analysis of HepG2 cells treated with CM WI-38 Primary, WI-38 Tumor, HCC-827, and HCC-4006 or left NT as a control. p53 protein was immunoprecipitated using p53-specific H47 polyclonal antibody (p53 antibody) or with beads only as a control. qRT-PCR was performed using specific primers against the p53 REs indicated in Fig. 2c. Values were normalized to 1% input of the corresponding sample. Results presented as mean ± SE (*t*-test, *P < 0.05, **P < 0.01, *n* = 3 independent experiments). **f**, **g** MS analysis was performed on CM from HepG2 sh-con/sh-p53 cells that were treated with CM collected from HCC-4006 cells. We further analyzed the secreted proteins that were significantly changed in p53-dependent manner treatment [(sh-con treated/sh-con non-treated) <0.75 or >1.3, *t*-test *P* < 0.05 and (sh-con treated/sh-p53 treated) or (fold change sh-con/fold change sh-p53) *t*-test *P* < 0.05, *n* = 3 independent experiments]. **f** The secreted proteins that were significantly dependent on p53 were affiliated by gene-card analysis tool (GeneAnalytics) to their GO biological annotations. Different GO annotations were grouped to major categories as presented in the graph (fold change are listed in Table S6, annotations are listed in Table S7). Each dot represents different secreted proteins, distributed by its relevant fold change (sh-con/sh-p53) (downregulated proteins <1, upregulated proteins >1). **g** Venn diagram presenting the biological GO annotations of proteins identified by MS analysis following all examined conditions (e.g., physiological conditions, Nutlin-3a and CM from HCC-4006 cells treatments). Full annotations are listed in Table S8. Blue represents unique annotations for p53 physiological conditions. Yellow represents unique annotations for Nutlin-3a treatment. Red represents unique annotations HCC-4006 CM treatment. Green represents shared annotations between p53 physiological conditions and Nutlin-3a treatment. Orange represents shared annotations between Nutlin-3a and HCC-4006 CM treatments. Purple represents shared annotations between all conditions

To further confirm the hypothesis that lung tumors are able to activate the hepatic p53, we utilized an in vitro model, where the effect of healthy and tumor-derived lung cells on hepatic p53 was compared. We used WI-38 primary embryonic lung fibroblast (WI-38 primary) that were genetically manipulated to generate transformed cell lines (WI-38 tumor line)^37^. To examine their effect on hepatic cells, sh-con/sh-p53 HepG2 cells were exposed to CM collected from this model. HepG2 sh-con cells treated with CM of WI-38 tumor line displayed higher expression levels of total and phosphorylated p53 protein when compared with their controls, indicating p53 accumulation and activation (Fig. 4a). Accordingly, the mRNA expression levels of p21, IGFBP1, SERPINE1, and SHBG were induced in a p53-dependent manner following treatment with CM from WI-38 tumor cells (Fig. 4b). To corroborate this effect, we used CM from additional cancer cell lines: HCC-827 and HCC-4006. Consistent with WI-38 system, the CM treatment yielded elevation in total and phosphorylated/acetylated HepG2 p53 (Fig. 4c), followed by upregulation of the p53 target genes (Fig. 4d). Next, we examined whether p53 binds to these genes' chromatin also upon treatment with various tumor cell-derived CM. ChIP analysis revealed an enrichment of p53 REs upon treatment with tumor-derived CM (Fig. 4e). In accordance with our in vivo observations, these results further suggest that lung tumors activate hepatic p53, which in turn leads to expression and secretion of its targets. In addition to lung tumors, we observed p53 activation in HepG2 treated with tumor-derived CM of other organs, suggesting that this effect is not lung-specific (Figure S6). To obtain a global view on this effect, we preformed MS analysis of CM from HepG2 sh-con/sh-p53 cells that were treated with CM from lung cancer cell line HCC-4006. Sixteen p53-dependent secreted proteins were detected (Table S6). While 23% out of them (3 secreted proteins) were shared with those that elevated with Nutlin-3a treatment, 77% (13 secreted proteins) were altered following CM treatment. Interestingly, by analyzing their functional annotations, we found that pathways related to metabolism were absent, while new pathways related to cells migration and adhesion were found (Fig. 4f, Table S7). To validate the effect on cell migration, we performed a wound healing assay, where migration of HCC-4006 cells were examined upon treatment with CM obtained from HepG2 sh-con/sh-p53 cells that were pretreated with CM from HCC-4006. We observed that cells treated with CM from HepG2 sh-con presented increased capability to close wound area (Figure S7A). This was accompanied with an elevation of well-accepted migratory markers (Figure S7B). Finally, to gain a global view on hepatic p53 role, we analyzed the biological pathways of the proteins secreted from HepG2 upon the different examined stimuli identified by MS analysis. We noticed that only 6% of the annotations were shared between all conditions, all of them related to injury response, suggesting that p53 regulates general injury response both under physiological and pathological conditions. However, only 16% of the annotations are unique for physiological conditions and all remaining appear upon p53 activation, suggesting that p53 activation allows additional p53-mediated functions. Moreover, the inducers by which p53 is activated contribute to its function diversity. Each treatment uniquely presents different annotations. While Nutlin-3a stimulus leads to metabolic processes, the HCC-4006 CM treatment leads to cells' adhesion, migration, and immune-response pathways. Notably, 20% of the annotations shared between Nutlin-3a and HCC-4006 CM treatments. These are mainly related to immune response and hemostasis, which are absent under p53 physiological conditions (Fig. 4g, Table S8). These findings imply that distinct insults including specific drugs or CM of cancer cell lines can activate hepatic p53, which in turn differently affects the expression of various liver-secreted proteins that are implicated in diverse biological functions.

## Discussion

In addition to the well-accepted notion that the tumor-suppressor p53 plays a central role in regulating cell-cycle arrest and apoptosis in response to various stress signals, recent data suggest that p53 also induce a variety of non-canonical pathways that affect the cell surroundings^1,2^. Data presented here shows that, upon exposure to several types of stress stimuli, the hepatic p53 acts in non-cell autonomous fashion by affecting the liver secretome. Examination of sera from WTp53 mice and p53 KO mice indicated significant differences in the levels of several hepatic enzymes essential for normal liver activity (Fig. 1a). Additionally, MS analysis of HepG2 cells secretome identified p53-dependent secreted factors which are involved in different pathways that are part of physiological liver functions such as injury response and lipid metabolism. Indeed, in addition to its role in damaged cells, p53 has basal activities related to homeostatic regulation of metabolic processes^38^. Notably, many of the secreted factors were found to be downregulated by p53 (Fig. 1b, c). This observation is in accordance with recent evidence suggesting that, under physiological conditions, p53 represses genes expression either by binding to their chromatin^39^ or indirectly by regulating the DREAM complex^40^. Interestingly, overexpression of several of the downregulated factors (e.g., APOA4, APOA5, APOE) were shown to be involved in various liver syndromes^41^. Therefore, it is important to keep the expression of these genes controlled for maintaining normal liver homeostasis. In addition to p53 basal functions, our data demonstrated that activation of p53 following stress leads to alterations in liver secretome profile by regulating the factors’ expression (Figs. 2a, b, e and 4a–d). As a transcription factor, p53 regulates its targets by directly binding to their chromatin via specific REs^2^. Indeed, we found that p53 binds to the secreted targets chromatin (Figs. 2c, d and 4e). Notably, the secretory machinery was not regulated by p53 (Table S3). Altogether, our data suggest that p53 induces the expression of the secreted proteins by binding to their chromatin, rather than regulating the entire secretory machinery.

Interestingly, while under physiological conditions p53 regulates processes related to normal liver functions, activated p53 governs diverse and unique pathways related to various liver metabolic processes. Moreover, p53 activation also led to the secretion of immune response, cell–cell adhesion, and ECM remodeling factors, which are related to SASP^42^. This adds level of complexity to the known p53 metabolic regulation^3^. As various chemotherapy agents and IR alter the p53-dependent secreted profile, our results imply intriguing cancer treatments and possible liver side effect relationship. Indeed, it was suggested that various drugs might induce liver failure^9,43^.

The liver is a prevalent site for metastasis of many tumor types, including the lung^44^. Additionally, lung tumors were shown to affect the liver metabolome^19^. These data suggest a possible liver–tumor crosstalk. Indeed, we showed here that the existence of lung tumors could activate hepatic p53, leading to higher levels of the secreted target, SERPINE1. Furthermore, we found that p53 KO mice harboring lung tumors are more susceptible to liver dysfunction compared to WTp53 mice. These findings agrees with our in vitro model in which lung tumors CM can activate p53 in HepG2 cells, which mediate secretion of factors associated with immune response, cell adhesion, and migration. Accordingly, we showed that CM obtained from WTp53 HepG2 cells, pretreated with CM from lung tumor cells, is able to induce tumor cell migration, implying that these secreted factors might support metastasis formation. The observed phenomenon that the hepatic WTp53 may support tumor cells' migration can be explained by the notion that, while some of p53 activities support liver homeostasis in the short term, these activities may lead to malignant transformation in the long term^45^. Apparently, these elevated factors are part of the SASP, which in this aspect may act as cancer-promoting proteins. Therefore, depending on the stimulus, members of the SASP that are secreted from the damaged liver in a p53-dependent manner can act either as mediators of homeostasis or as cancer promoting, as was also suggested before^42^. While WTp53-driven senescent cells can inhibit tumor progression and keep healthy tissue^16,17,46,47^, senescent phenotype can also contribute to tumor development and metastasis^48,49^. Accordingly, some of the p53-dependent secreted factors show this dichotomy as well. One example is the SERPINE2, which can lead to metastasis or apoptosis of cancer cells under different conditions^50–53^. Additional p53 target, CTSB, regulates fibrosis and inflammation and attenuates tumor development following liver injury^54,55^. Yet, CTSB was also found to support tumor progression in many cancer types, such as breast, lung, and more^56^. In this study, we suggest a p53-dependent inter-organ crosstalk, in which distal tumors in the lung signal the liver, which in response activates p53 to secrete various proteins that might affect the whole-body homeostasis. It has been proposed that cancer behaves as a systemic dictator that interacts with different tissues to control their metabolism^57^ and influence the body macroenvironment by releasing soluble factors into the blood or lymph vessels^58^. Altogether, our data broaden our knowledge regarding the non-canonical functions of WTp53, which leads to the elevation of various hepatic-secreted factors that are important for normal liver functions. Moreover, in cancer patients, where homeostasis is disrupted owing to the tumor growth and treatments, the hepatic p53 is activated and leads to alteration in the liver secretome. This might be important for maintaining liver functionality but in addition may give rise to side effects and cancer aggressiveness. Thus our data suggest additional role for p53 as a central regulator of whole-organism homeostasis both under physiological and pathological conditions.

## Materials and methods

### Cell culture

All cell lines were cultured in a humidified incubator at 37 °C and 5% CO_2_. HepG2 cells were kindly provided by Professor Yehiel Zick. HCC827, HCC4006, BT474, HT29, LS411N, and PC9 cells were kindly provided by Dr. Ravid Straussman. HepG2, HCC827, HCC4006, BT474, HT29, LS411N, and PC9 cells were cultured in Dulbecco’s modified Eagle’s medium (DMEM) supplemented with 10% fetal calf serum (FCS), 2 mM L-Glutamine, 1 mM sodium pyruvate, and 100 mg/ml penicillin/streptomycin (Biological Industries, Beit-Haemek, Israel). D122 cells were kindly provided by Professor Lea Eisenbach and maintained as described^36^. Primary WI-38 cells were purchased from ATTC and transformed WI-38 cells were generated and maintained as described^37^.

### Retroviral infections

WTp53 was stably knocked down by retroviral infections as previously described^5^.

### RNA isolation and quantitative real-time PCR

RNA isolation from cell lines and quantitative real-time PCR (qRT-PCR) was conducted and analyzed as previously described^5^. The specific primers used for qRT-PCR are listed in Table S8. Total RNA from mice liver tissues was isolated using the Direct-zol RNA MiniPrep Kit (Zymo Research, CA, USA). A 2 µg aliquot of the total RNA was reverse transcribed into cDNA and qRT-PCR was performed as described^5^. The specific primers used for qRT-PCR are listed in Table S9.

### Western blot

Cells were lysed in Tris Triton Lysis Buffer (50 mM Tris-HCl, 100 mM NaCl, 1% Triton X-100, 0.5% sodium deoxycholate, 0.1% sodium dodecyl sulfate (SDS)) supplemented with Protease Inhibitor Cocktail (Sigma-Aldrich, Rehovot, Israel) for 15 min on ice and centrifuged for 15 min. Supernatants were analyzed for protein concentration by BCA reagent (Thermo-Scientific, NY, USA). Protein extracts (50 µg) were boiled in sample buffer [140 mM Tris (pH 6.8), 22.4% glycerol, 6% SDS, 10% β-mercaptoethanol, and 0.02% bromphenol blue] and loaded on 10–12% SDS-polyacrylamide gel. Proteins were transferred to a nitrocellulose membrane at semi-dry conditions.

The following primary antibodies were used: α-p21 (sc-397 Santa Cruz Biotechnology, TX, USA), α-Phospho-p53 (Ser15) and α-Acetyl-p53 (Lys382) (Cell Signaling, MA, USA), α-SERPINE1 (MA5-Cell 17171, Thermo scientific, NY, USA), α-IGFBP1 (ab181141, abcam, Cambridge, UK), α-GAPDH (mab374, EMD Miilipore, MA, USA), α-p53 (DO-1; kindly provided by Professor David Lane), and goat polyclonal α-p53 horseradish peroxidase-conjugated (Minneapolis, MN, USA). The protein–antibody complexes were detected by horseradish peroxidase-conjugated secondary antibodies: anti-mouse or goat anti-rabbit (Jackson Immunoresearch Laboratories, PA, USA) and the ECL Kit (Thermo Scientific) and analyzed by ChemiDoc MP imaging system (BIO-RAD, CA, USA).

### ChIP assay

ChIP was done as previously described^5^.

Samples were precleared by incubating with blocked protein A beads (Santa Cruz Biotechnology, TX, USA) for 2 h at 4 °C. The precleared chromatin was mixed by rotation for 12 h at 4 °C with blocked protein A beads and 1 µg of polyclonal h47 α-p53 (produced in our laboratory) or with beads only as a control. DNA samples were extracted using the QIAquick PCR Purification Kit (Qiagen, Hilden, Germany). qRT-PCR was performed as described above with each sample containing 2 µl of immunoprecipitated DNA. Values were normalized for 1% input values. The specific primers surrounding p53 RE used for qRT-PCR are listed in Table S10.

### Chemicals compounds

The following agents were used: Nutlin-3a (Alexis-Biochemical, CA, USA), 5-fluorouracil, CCl_4_, corn oil (Sigma-Aldrich, Rehovot, Israel), and Oxaliplatin (LC Laboratories, MA, USA).

### CM assay

WI-38 primary/tumor lines, HCC-827 and HCC-4006, were grown in their medium (as mentioned above). After 24 h, their medium was collected, filtered, and transferred to HepG2 sh-con/sh-p53 cells with ratio of 1:3 (fresh medium to CM). The HepG2 cells were grown with the CM for additional 24 h and then were collected and analyzed.

### Mass spectrometry

The MS-based proteomics was performed by Dr. Meital Kupervaser at the de Botton Institute for Protein Profiling, The Nancy and Stephen Grand Israel National Center for Personalized Medicine, Weizmann Institute of Science. Briefly, HepG2 sh-con/sh-p53 cells were subjected to the following treatments: Nutlin-3a and CM from HCC-4006 cells (as mentioned above) or remain un-treated as a control. After 18 h, their medium was washed and the cells were grown for additional 6 h with DMEM medium-free FCS and phenol red. The media was collected and concentrated using a 3 kDa MWCO filter to a final volume of 0.5 ml with protein concentration of 1.5–1.8 µg/µl and then subjected to a tryptic digest. The resulting peptides were analyzed on the liquid chromatography-MS instrument and the raw data were processed with the Genedata’s Expressionist software. The software used two search engines to identify the peptides in the sample, mascot and MSGF+, against the uniprot human proteome appended with 120 common contaminants. Data were normalized based on the total ion current. Protein abundance was obtained by summing the three most intense, unique peptides per protein, and after logarithmic transformation was used to identify significant differences across the biological replica. For further information, see Supplemental Experimental Procedures (Mass-Spectrometry). Fold changes and Student’s *t*-test were calculated based on the ratio of the treatment vs. control samples. For each treatment, we filtered for the proteins whose expression was altered upon treatment in p53 sh-con cells [(sh-con treated/sh-con non-treated) <0.75 or >1.3, *t*-test *P* < 0.05] and then we included only the proteins that were significantly induced in p53-expressing cells ((sh-con treated/sh-p53 treated) or (fold change sh-con/fold change sh-p53) *t*-test *P* < 0.05. *n* = 3 independent experiments).

Biological GO annotation analysis was done using gene-card analysis tool, GeneAnalytics^22^.

### Wound-healing assay

HepG2 sh-con/sh-p53 cells were treated with CM from HCC-4006 or left un-treated as a control. After 18 h, their medium was washed and the HepG2 sh-con/sh-p53 cells were grown for additional 6 h with medium-free FCS and phenol red. This media was collected for further wound-healing analysis as follows: HCC-4006 cells were seeding on Ibidi culture inserts for 24 h. Following insert removal, HCC-4006 cells were treated with HepG2 CM collected as mentioned above. HCC-4006 cells' migration was recorded at 0 h and 24 h posttreatment by operetta High-Content Imaging System (PerkinElmer). Quantification of the wound area was performed using the NIH ImageJ software (http://rsb.info.nih.gov/ij/).

### Mice

WTp53 and p53 KO C57BL/6 mice were provided by Guillermina Lozano, MD Anderson Cancer Center, Houston)^20^. Experiments were performed in accordance with Weizmann Institute of Science regulations for institutional animal care and use committees. All the experiments were done on male mice aged 8 weeks.

### Blood tests

The collected mice blood samples were centrifuged at 14,000 rpm for 15 min. The mice sera was collected and analyzed for the presence of circulating enzymes by diagnostic veterinary pathology services (PathoVet Ltd, Kfar Bilu B, Israel and AML Ltd, Herzliya, Israel).

### CCl_4_

Mice were injected intraperitoneally with 1 ml/kg of 10% CCl_4_ diluted in corn oil. Injection of corn oil alone served as a control. Forty eight hours after the experiment, mice were sacrificed and analyzed.

### Irradiation

Mice received total-body IR delivered by X-RAD 320 IX irradiator at 10 Gy. Twenty four hours after the experiment, mice were sacrificed and analyzed.

### Lung tumor formation and staining

D122 cells^36^ were collected, re-suspended in phosphate-buffered saline (PBS), and injected intravenously into C57BL/6J p53 WT/KO mice (10^6^ cells/50 µl). Injection of PBS alone served as a control. Lung tumors were allowed to be established for 7 days after injection, and mice were sacrificed and analyzed. Mice lungs were fixed in 4% paraformaldehyde, decalcified, and embedded in paraffin blocks. Sections were stained with hematoxylin and eosin (H&E). The designation of a tumor's presence was based on histological criteria.

### Immunohistochemistry

Paraffin sections were de-paraffinized and antigen-retrieved (in 10 nM citric acid pH 6). The sections were incubated in PBS solution containing 20% normal horse serum and 0.2% Triton for 1 h and then incubated overnight with α-SERPINE1 (MA5-17171, Thermo scientific, NY, USA). Sections were labeled with diaminobenzidine using the ABC Kit (Vector, Burlingame, CA, USA). Additionally, sections were stained by H&E. Stained sections were scanned, and digital images were collected and analyzed using the QuPath software^59^.

## Electronic supplementary material

Supplemental material
