# Supplementary material for: Various stress stimuli rewire the profile of liver secretome in a p53-dependent manner
Source: Cell Death Dis. 2018 May 29;9(6):647. doi: 10.1038/s41419-018-0697-4 (PMC5974134; doi:10.1038/s41419-018-0697-4)
Supplement: Supplementary file 1 — Supplemental material [file 41419_2018_697_MOESM1_ESM.docx]

**Figure S1- p53 is downregulated in HepG2 cells harboring sh-p53 construct.**


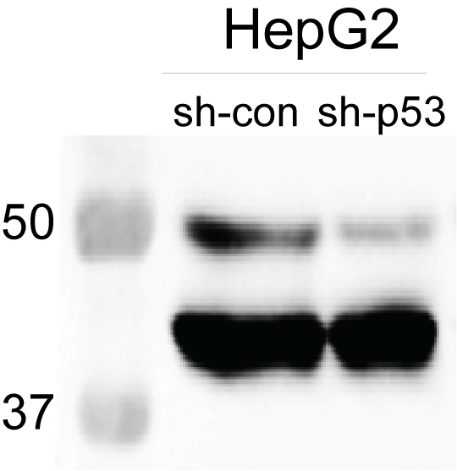


p53

GAPDH

HepG2 cells that endogenously express WTp53, were stably introduced with shRNA against p53 (sh-p53) or non-relevant sh-RNA as a control (HepG2 sh-con). Protein levels of p53 were measured by Western blot. β-Actin was used as loading control. The blot is representative of at least three experiments.

**Figure S2- Livers of WTp53 and p53 KO mice following D122 injection remained intact without neoplastic cells.**

WTp53+D122 injection (7 days)


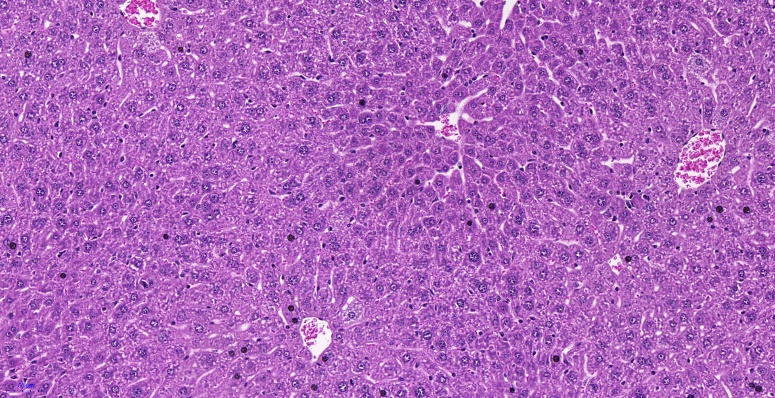


p53 KO+D122 injection (7 days)


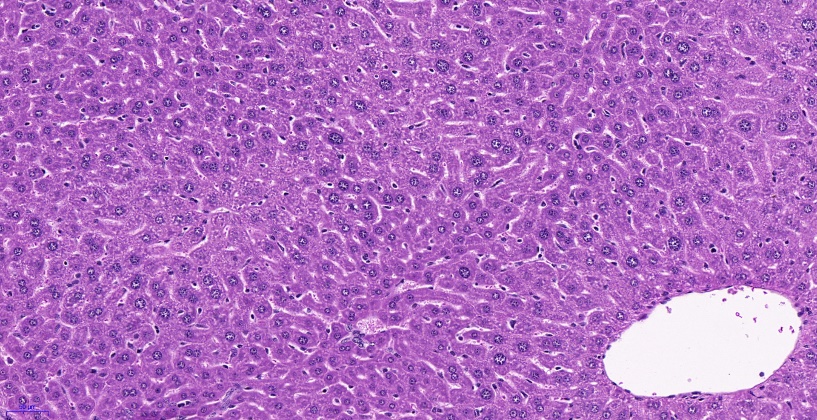


Liver sections were stained with H&E. The photo is representative of 4 individual mice (WTp53 treated n=4 mice, p53 KO treated n=4 mice).

**Figure S3- SERPINE1 protein levels are elevated in livers of WTp53 mice following lung tumor development in a p53-dependent manner.**

**
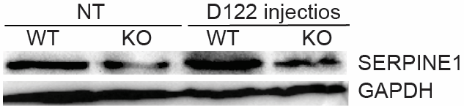
**

WTp53 and p53 KO mice were intravenously injected with D122 cancer cells (10^6^ cells) or with PBS alone as a control (NT). After 7 days lung tumors were generated and mice were sacrificed. Proteins were extracted from mice livers, and the levels of SERPINE1 and GAPDH (as a loading control) were measured by western blot. Each band represents a pool of 4 mice livers extract.

**Figure S4- the expression of SERPINE1 is higher in livers of WTp53 mice compared to p53 KO mice following lung tumor presence.**


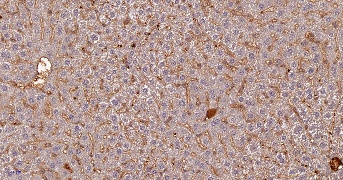

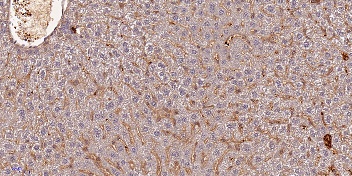

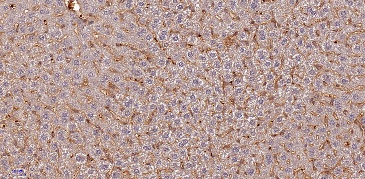

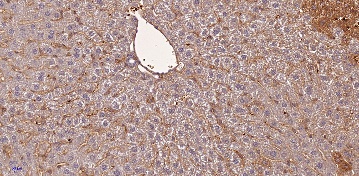
WTp53 NT


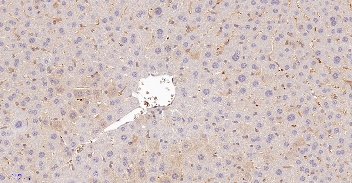

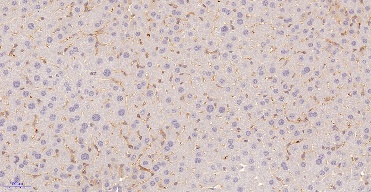

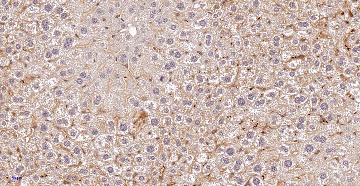

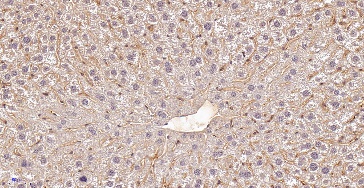
p53 KO NT


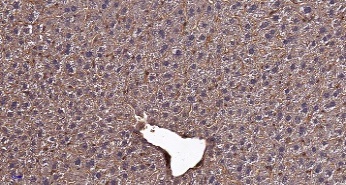

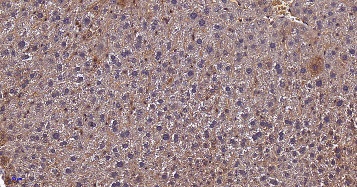

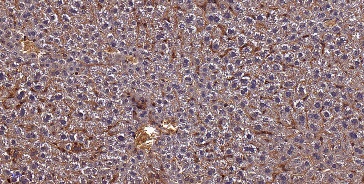

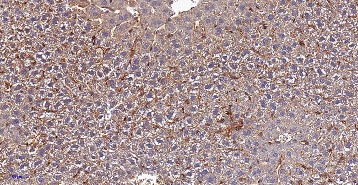
WTp53+D122 injection (7 days)


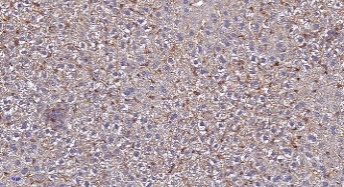

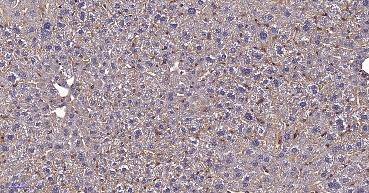

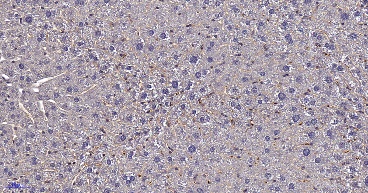

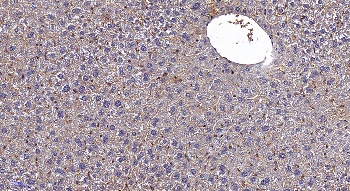
p53 KO+D122 injection (7 days)

Liver sections were immunohistochemically stained with an anti-SERPINE1 antibody combined to a biotinylated secondary antibody (brown; DAB). This figure presents additional photos of the staining depicted in Figure 3E. WTp53 NT n=4 mice, p53 KO NT n=4 mice, WTp53 treated n=4 mice, p53 KO treated n=4 mice).

**Figure S5- p53 RNA levels do not altered following p53 activation**

HepG2 (sh-con/sh-p53) were treated with CM of different cell lines (WI-38 Primary, WI-38 Tumor, HCC-827, HCC-4006 or remain non-treated as a control. RNA levels of p53 were measured by qRT- PCR. Results presented as mean ± SE, n=3 independent experiments.

**Figure S6- Conditioned media derived from different human cell lines induce hepatic p53 activation.**

**
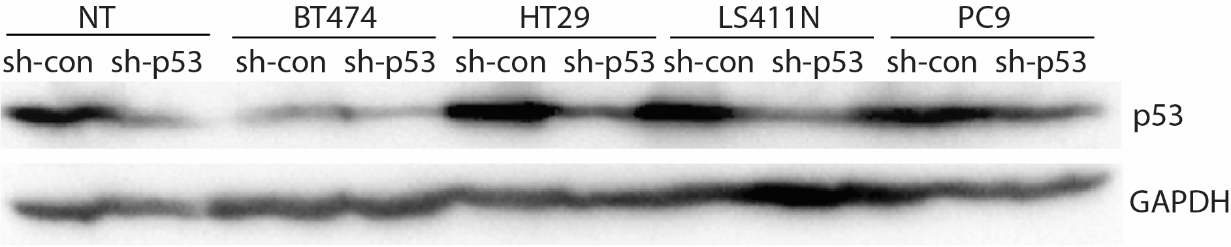
A.**


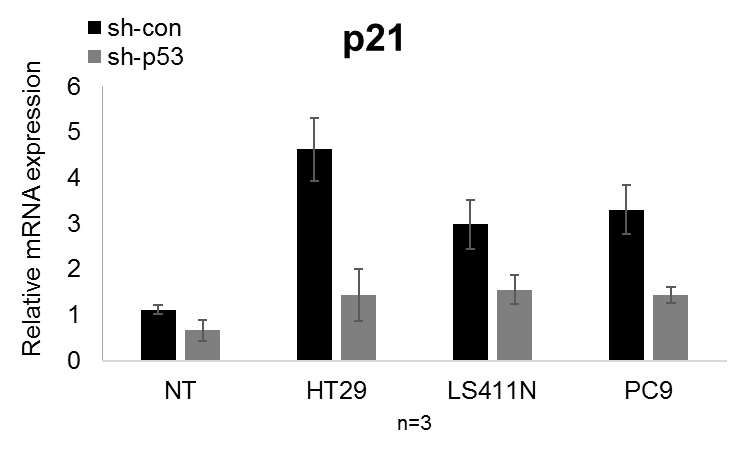


**B.**

**

**

*

HepG2 sh-con/sh-p53 cells were supplemented with CM collected from different human cell lines, BT474 (breast), HT29 (colon), LS411N (cecum) and PC9 (lung) or remained non-treated (NT). After 24 hours the treated HepG2 cells were collected. (A) Protein levels of p53 were measured by Western blot. GAPDH was used as loading control. (B) mRNA levels of *p21* was measured by qRT-PCR analysis. Results presented as mean ± SE. *t*-test, *P <0.05, **P <0.01. n=3 independent experiments.

**Figure S7- migration of HCC4006 cells following HepG2 sh-p53/sh-con CM treatment**

**A**


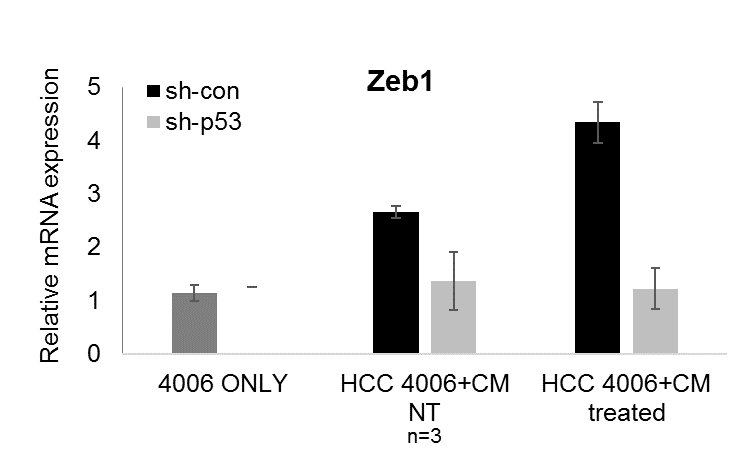


******

**B**


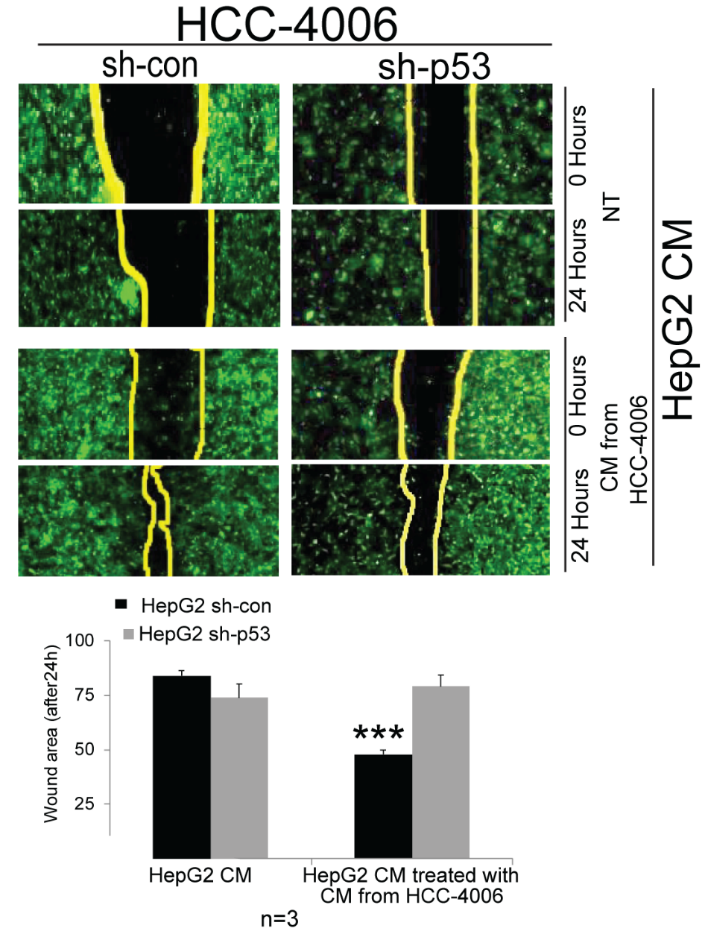


******


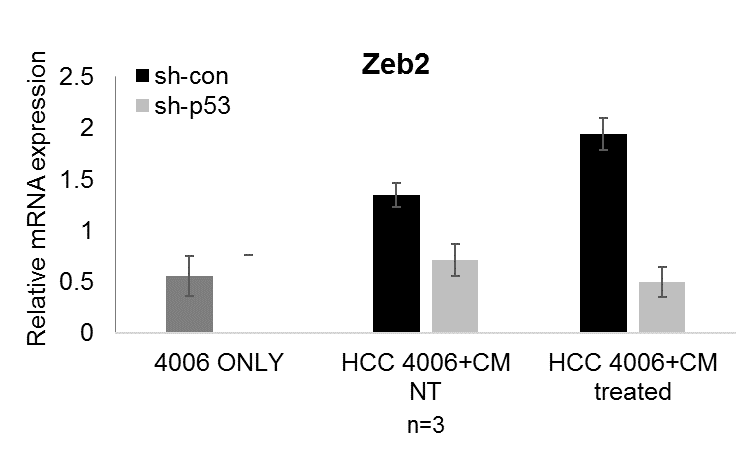


*****

*****


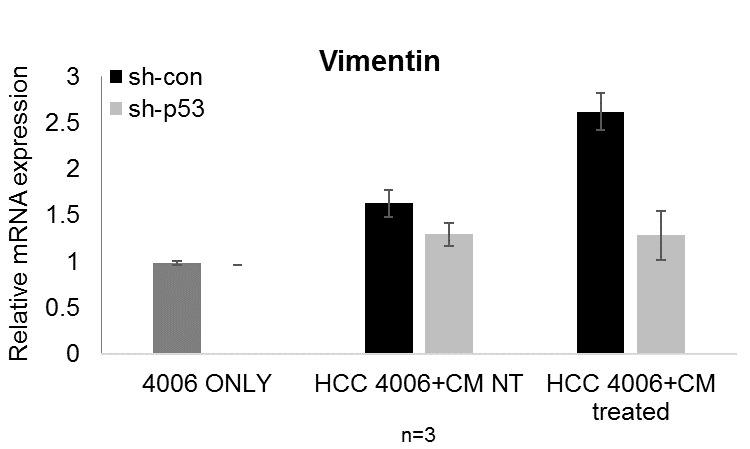


*****

*****

A. HCC-4006 cells were seeding on Ibidi culture inserts for 24 hours. Following insert removal, HCC-4006 cells were treated with CM of HepG2 sh-con/sh-p53 that were pre-treated with or without CM of HCC-4006. HCC-4006 cells migration was recorded at 0h and 24h post treatment. Upper panel- representative wound images of at least three independent experiments. Yellow lines indicate the wound borders. Lower panel-quantification of the wound area which was expressed as percentage of the wound area (maximum 100%). In each condition, the percentage is related to the wound area at time 0 hours. Results presented as mean ± SE. t-test, ***P <0.001. n=3 independent experiments.

B. HCC-4006 cells that were treated with CM of HepG2 sh-con/sh-p53, pre-treated with or without CM of HCC-4006 (see A) were analyzed for mRNA expression of several migration markers (ZEB1, ZEB2 and Vimentin) by qRT-PCR. Results presented as mean ± SE. t-test, *P <0.05, **P <0.01. n=3 independent experiments.

**Table S1- list of p53 dependent secreted proteins in CM of HepG2 cells (p53 physiological conditions)**

| **Protein name** | **Fold change (sh-con/ sh-p53 )** |
| --- | --- |
| NPNT | 1.71502E-06 |
| GLUL | 6.66036E-06 |
| PLA2G2A | 0.0243178 |
| FSTL3 | 0.096631332 |
| SMARCC1 | 0.121035505 |
| DKK1 | 0.123958333 |
| VCAN | 0.176998064 |
| HLA | 0.188974638 |
| NPTXR | 0.198180958 |
| SF3B4 | 0.198567577 |
| CHMP4B | 0.20695206 |
| RTFDC1 | 0.222635432 |
| SORT1 | 0.227308858 |
| NBL1 | 0.230515616 |
| HSPB1 | 0.268662787 |
| NRCAM | 0.271409933 |
| DKK4 | 0.27353991 |
| PLAU | 0.299195833 |
| ACO2 | 0.323931947 |
| GPC3 | 0.32425 |
| CALD1 | 0.351169358 |
| CTNNBIP1 | 0.373049678 |
| RPS28 | 0.384137879 |
| FSCN1 | 0.386160435 |
| LMNA | 0.38927571 |
| STMN1 | 0.401612917 |
| SDC4 | 0.405903393 |
| CD109 | 0.422197584 |
| S100A11 | 0.43273564 |
| HSP90AB4P | 0.43502273 |
| APOA5 | 0.438480439 |
| MT2A | 0.441427463 |
| FBLN1 | 0.4562482 |
| ACSL4 | 0.460228547 |
| IGFBP4 | 0.468960168 |
| RARRES2 | 0.474831991 |
| TES | 0.475302189 |
| UXS1 | 0.489804916 |
| HEXA | 0.4907574 |
| TPP1 | 0.510018345 |
| PSAP | 0.511302373 |
| PPT1 | 0.515145986 |
| FUBP1 | 0.515207809 |
| FGB | 0.529563492 |
| NPC2 | 0.543890088 |
| DKFZp686J1372 | 0.545211172 |
| YWHAE | 0.551020408 |
| CKAP5 | 0.570910596 |
| HEXB | 0.576915085 |
| TGOLN2 | 0.579042273 |
| RELN | 0.580737705 |
| HAGH | 0.595529205 |
| HLA | 0.601117422 |
| HSPA13 | 0.606700771 |
| CLSTN1 | 0.612769486 |
| IFI30 | 0.647968943 |
| JAG1 | 0.666182315 |
| FLNA | 0.673290356 |
| PTGES3 | 0.679019193 |
| CTSA | 0.698333534 |
| SEMA7A | 0.700051969 |
| SF3B3 | 0.704359673 |
| CDH2 | 0.730185497 |
| APOE | 1.320143885 |
| CCL15 | 1.342477765 |
| C1S | 1.691708452 |
| MROH7 | 1.701279455 |
| KIF2B | 1.883538592 |
| C8B | 1.943229898 |
| VNN1 | 2.117653497 |
| SPARC | 2.411005405 |
| APOA4 | 2.519944372 |
| CTGF | 2.844806008 |
| FST | 4.068963778 |
| SULF2 | 1667411.125 |

MS analysis was performed on CM from HepG2 sh-con/sh-p53 cells. The analysis refers to secreted proteins that were significantly dependent on p53 [(sh-con/sh-p53) <1.3 or >1.3, *t*-test P <0.05, n=3 independent experiments].

| **GO biological annotations**  Development | | **Relevant proteins** |
| --- | --- | --- |
|  | Ossification | CTGF, FSTL3, SORT1, SPARC |
|  | Neuron Projection Regeneration | APOA4, APOA5, APOE |
|  | Lung Development | CTGF, FSTL3, GPC3, SPARC |
|  | Cell Morphogenesis Involved in Differentiation | DKK1, RELN |
|  | Central Nervous System Development | NRCAM, VCAN, RELN, TPP1 |
|  | Cerebral Cortex Development | FLNA, YWHAE, RELN, CDH2 |
|  | Negative Regulation of Canonical Wnt Signaling Pathway | APOE, IGFBP4, DKK1, DKK4, GPC3, CDH2 |
|  | Establishment or Maintenance of Microtubule Cytoskeleton Polarity  Cytoskeleton | CKAP5, LMNA |
|  | Microtubule Depolymerization | STMN1, KIF2B |
|  | Negative Regulation of Activin Receptor Signaling Pathway | FST, FSTL3 |
|  | Regulation of Axon Extension | APOE, NRCAM |
|  | Post-translational Protein Modification  Miscellaneous signaling | APOA5, APOE, IGFBP4, VCAN, FSTL3, TGOLN2, GPC3, CDH2 |
|  | Lysosome Organization | PPT1, HEXB, TPP1 |
|  | Regulation of Autophagy | CHMP4B, PSAP, HSPB1 |
|  | Positive Regulation of ERK1 and ERK2 Cascade | NPNT, SEMA7A, FGB, CTGF, PLA2G2A, CCL15 |
|  | Blood Coagulation, Fibrin Clot Formation  Injury response | FBLN1, FGB |
|  | Plasminogen Activation | FGB, PLAU |
|  | Platelet Aggregation | FGB, FLNA, HSPB1 |
|  | Platelet Degranulation | PSAP, FGB, FLNA, RARRES2, CD109, SPARC |
|  | Cholesterol Efflux | APOA4, APOA5, APOE, NPC2 |
|  | Chylomicron Assembly | APOA4,APOE |
|  | Glycosaminoglycan Biosynthetic Process | PSAP, CTSA, HEXB, HEXA,FGB, FLNA, RARRES2, CD109, SPARC,SDC4, VCAN,GPC3 |
|  | High-density Lipoprotein Particle Assembly | APOA4, APOA5, APOE,PLA2G2A |
|  | Retinoid Metabolic Process  Metabolism processes | APOA4, SDC4, APOE, RARRES2, GPC3 |
|  | Chondroitin Sulfate Catabolic Process | VCAN, HEXB, HEXA |
|  | Keratan Sulfate Catabolic Process | HEXB, HEXA |
|  | Lipid Metabolic Process | APOA4, APOA5, APOE,PASP |
|  | NMDA Glutamate Receptor Clustering | APOE, RELN |
|  | Phosphatidylcholine Metabolic Process | APOA4, APOA5 |
|  | Positive Regulation of Fatty Acid Biosynthetic Process | APOA4, APOA5 |
|  | Triglyceride Homeostasis | APOA4, APOA5, APOE,ACSL4 |
| ECM | Extracellular Matrix Organization | NPNT, FBLN1, FGB, VCAN, SPARC |

**Table S2: Go biological annotations of p53 dependent secreted proteins**

| **Protein name** | **Fold change physiological conditions** | **Fold change**  **Nutlin** | **Fold change CM HCC-4006** | **Secretion machinery pathways** |
| --- | --- | --- | --- | --- |
| ALB | 0.968785 | 1.156502 | 0.980437 | Vesicle-mediated Transport, Binding and Uptake of Ligand |
| ACTR2 | 0.927172 | 0.823036 | 0.954354 | Vesicle-mediated Transport, Clathrin-mediated Endocytosis |
| ARPC4 | 0.82446 | 0.960392 | 0.943122 | Vesicle-mediated Transport, Clathrin-mediated Endocytosis |
| APOB | 0.933242 | 0.819547 | 0.99058 | Vesicle-mediated Transport, Clathrin-mediated Endocytosis, Binding and Uptake of Ligand |
| IGF2R | 0.902123 | 1.125811 | 1.053589 | Vesicle-mediated Transport, Clathrin-mediated Endocytosis, Clathrin Derived Vesicle Budding |
| MAN1A1 | 1.06255 | 0.861132 | 0.948679 | Vesicle-mediated Transport |
| GALNT2 | 0.85302 | 0.874511 | 1.081346 | Vesicle-mediated Transport, Golgi-to-ER Retrograde Traffic |
| COL3A1 | 1.13166 | 0.978118 | 0.946123 | Vesicle-mediated Transport, Binding and Uptake of Ligand |
| GALNT1 | 0.877103 | 0.945281 | 1.081346 | Vesicle-mediated Transport, Golgi-to-ER Retrograde Traffic |
| HPX | 0.89048 | 0.897558 | 1.133136 | Vesicle-mediated Transport, Binding and Uptake of Ligand |
| RAB5C | 0.824527 | 0.946818 | 0.946036 | Vesicle-mediated Transport, Clathrin-mediated Endocytosis, Clathrin Derived Vesicle Budding |
| NEDD8 | 0.964774 | 0.942258 | 1.020329 | Vesicle-mediated Transport, Clathrin-mediated Endocytosis |
| AMBP | 0.896373 | 0.993256 | 0.869942 | Vesicle-mediated Transport, Binding and Uptake of Ligand |
| TFG | 0.817279 | 1.039166 | 0.987089 | Vesicle-mediated Transport, Clathrin-mediated Endocytosis |
| TOR1B | 1.044096 | 0.824056 | 1.113271 | Vesicle-mediated Transport |
| GFPT1 | 0.830427 | 0.954559 | 0.997985 | Transport to The Golgi and Subsequent Modification |
| MGAT5 | 1.196911 | 1.008065 | 0.8 | Transport to The Golgi and Subsequent Modification |
| UGGT1 | 0.993054 | 1.0438 | 1.134029 | Transport to The Golgi and Subsequent Modification |
| ST6GAL1 | 0.915053 | 1.040549 | 0.929008 | Transport to The Golgi and Subsequent Modification |

**Table S3- list secreted proteins in CM of HepG2 cells** related to the secretion machinery

Fold change of physiological conditions (HepG2 sh-con vs. HepG2 sh-p53)

Fold change of Nutlin-3a and HCC 4006 CM (HepG2 sh-con treated vs. HepG2 sh-con NT)

**Table S4- list of p53 dependent secreted proteins in CM of HepG2 cells upon Nutlin-3a treatment**

| **Protein name** | **Fold change (sh-con NT/sh-con treated )** |
| --- | --- |
| LCAT | 1.55282E-06 |
| HSPE1 | 0.188406144 |
| DKK1 | 0.214478529 |
| ADAMTSL2 | 0.231674333 |
| COCH | 0.303926123 |
| GLA | 0.344691897 |
| LGMN | 0.369906103 |
| CPB2 | 0.403242821 |
| RARRES2 | 0.405113141 |
| PVR | 0.416763638 |
| NID1 | 0.421159715 |
| FUCA2 | 0.447206395 |
| CPN2 | 0.474654618 |
| NPC2 | 0.502060062 |
| SLC39A14 | 0.502076998 |
| IFI30 | 0.505852156 |
| APOA1 | 0.516666667 |
| PPT1 | 0.519859628 |
| CLU | 0.537534247 |
| VASN | 0.53913946 |
| CDH2 | 0.543974296 |
| ORM2 | 0.552199851 |
| SMOC1 | 0.560971402 |
| APOA2 | 0.589409985 |
| ORM1 | 0.591066282 |
| FBLN1 | 0.639351312 |
| CTSA | 0.671670898 |
| B4GALT1 | 0.679603322 |
| C8B | 0.683031235 |
| PROS1 | 0.691045635 |
| LSM2 | 0.692948091 |
| TARS | 0.698213304 |
| NOTUM | 0.701107011 |
| CFI | 0.709583029 |
| SDF4 | 0.710930574 |
| PTPRK | 0.711659784 |
| NEU1 | 0.727614217 |
| BTD | 0.729165339 |
| TF | 0.736620312 |
| LSR | 0.739934367 |
| MAT2B | 0.741235187 |
| SPARC | 0.75 |
| NUCB1 | 0.754867909 |
| DSC2 | 0.757543711 |
| F11 | 1.304517869 |
| SDC4 | 1.386438968 |
| PFDN2 | 1.415274995 |
| FKBP1A | 1.485072674 |
| TPP2 | 1.486869196 |
| IGFBP2 | 1.496026607 |
| SERPINA6 | 1.52145749 |
| CTSB | 1.593369197 |
| CCL15 | 1.616889082 |
| SERPINA3 | 1.623900951 |
| ITM2B | 1.624365482 |
| NPEPPS | 1.630730691 |
| HSPG2 | 1.682615904 |
| C4B | 1.944536151 |
| MATN3 | 2.18697479 |
| SERPINE1 | 2.193890123 |
| TINAGL1 | 2.204477261 |
| GPC1 | 2.218521746 |
| SAA4 | 2.234491946 |
| TIMP1 | 2.305683564 |
| AGRN | 2.325837106 |
| TIGAR | 2.409669944 |
| IGFBP1 | 2.522088353 |
| SEMA3F | 3.036193546 |
| SHBG | 3.185716243 |
| GDF15 | 3.269543974 |
| CTGF | 4.874615046 |
| LOXL4 | 6.272476928 |
| SULF2 | 6.958183154 |
| SERPINE2 | 8.899895723 |
| LCN15 | 11.62239781 |
| PLTP | 12.70202584 |
| DSC3 | 25.16507898 |
| CEL | 29.52312057 |

The analysis refers to secreted proteins that were significantly changed in p53 dependent manner upon Nutlin-3a treatment [(sh-con treated/sh-con non-treated) <1.3 or >1.3, *t*-test P <0.05 and (sh-con treated/sh-p53 treated), or (fold change sh-con/fold change sh-p53) *t*-test P <0.05 n=3 independent experiments]

**Table S5:**

**Go biological annotations of p53 dependent secreted proteins altered upon Nutlin-3a treatment**

| **GO biological annotations**  Metabolism processes | | **Relevant proteins** |
| --- | --- | --- |
|  | Cellular Protein Metabolic Process | APOA1, APOA2, CEL, FUCA2, HSPG2, IGFBP1,IGFBP2, ITM2B, MATN3, NOTUM, NUCB1, TF, TIMP1 |
|  | Cholesterol | APOA1, APOA2, CLU, LCAT, NPC2 |
|  | Chylomicron Assembly | APOA1, APOA2 |
|  | Glycosaminoglycan Biosynthetic Process | AGRN, APOA1, APOA2, GPC1, HSPG2, RARRES2, SDC4 |
|  | Lipoprotein Particle Assembly | APOA1, APOA2, LCAT, LSR |
|  | Retinoid Metabolic Process | AGRN, APOA1, APOA2, GPC1, HSPG2, RARRES2, SDC4 |
|  | Lipid Metabolic Process | APOA1, APOA2, CEL, CLU, HSPG2, LCAT, LCN15, LSR, NEU1, NPC2, PLTP |
|  | Regulation of Insulin-like Growth Factor | IGFBP1, IGFBP2 |
|  | Glycoside Catabolic Process | FUCA2, GLA |
|  | Glucocorticoid Metabolic Process | APOA1, SERPINA6 |
|  | Phosphatidylcholine Biosynthetic Process | APOA1, APOA2, LCAT |
|  | Platelet Degranulation  Injury response | APOA1, CLU, ORM1, ORM2, PROS1, RARRES2, SERPINA3, SERPINE1, SPARC, TF, TIMP1 |
|  | Regulation of Blood Coagulation | PROS1, SERPINE1, SERPINE2 |
|  | Hemostasis | CPB2, F11, PROS1 |
|  | Regulation of Fibrinolysis | PROS1, SERPINE1, SERPINE2 |
|  | Regulation of Plasminogen Activation | CPB2, SERPINE1, SERPINE2 |
|  | Acute-phase Response | ORM1, ORM2, SAA4, SERPINA3 |
|  | Neutrophil Degranulation  Immune response | B4GALT1, CTSA, CTSB, FUCA2, GLA, NEU1, NPC2, ORM1, ORM2, SERPINA3 |
|  | Acute Inflammatory Response | APOA2, B4GALT1 |
|  | Wound Healing | B4GALT1, SDC4, SPARC, TIMP1 |
|  | Regulation of Immune System Process | ORM1, ORM2 |
|  | Regulation of Cytokine Secretion Involved in Immune Response | APOA1, APOA2 |
|  | Regulation of Complement Activation | CFI, PROS1, CLU, CPB2, CPN2, C4B, C8B |
|  | Protein Oxidation | APOA1, APOA2 |
|  | Proteolysis  Miscellaneous signaling | ADAMTSL2, C4B, CFI, CPB2, CPN2, CTSA, CTSB, F11, LGMN, NPEPPS, TINAGL1, TPP2 |
|  | Receptor-mediated Endocytosis | APOA1, CFI, HSPG2, LOXL4, LSR, NPC2, PPT1, SPARC, TINAGL1 |
|  | Growth Factor Beta Receptor Signaling Pathway | APOA1, FKBP1A, GDF15, PTPRK |
|  | Peptidyl-methionine Modification | APOA1, APOA2 |
|  | Regulation of Tau-protein Kinase Activity | CLU, DKK1 |
|  | Regulation of Fibroblast Growth Factor Receptor | GPC1, SULF2 |
|  | Regulation of Endopeptidase Activity | C4B, PROS1, SERPINA3, SERPINA6, SERPINE1, SERPINE2, TIMP1 |
|  | Response to Peptide Hormone | CTGF, SPARC, TIMP1 |
|  | Regulation of Canonical Wnt Signaling Pathway | CDH2, DKK1, IGFBP1, IGFBP2, NOTUM |
|  | Post-translational Protein Modification | APOA1, APOA2, CDH2, FUCA2, IGFBP1, MATN3 , NOTUM, NUCB1, TF, TIMP1 |
|  | Extracellular Matrix Organization  ECM | ADAMTSL2 ,AGRN, B4GALT1, FBLN1, HSPG2, MATN3 , SERPINE1, SPARC |
|  | Regulation of Stress Fiber Assembly | APOA1, CTGF, SDC4 |
| Cell motility | Cell Adhesion | B4GALT1, CDH2, CTGF, DSC2, DSC3, PTPRK, PVR, TINAGL1 |

**Table S6- list of p53 dependent secreted proteins in CM of HepG2 cells that were treated with CM from HCC-4006 cells**

| **Protein name** | **Fold change (sh-con NT/sh-con treated )** |
| --- | --- |
| GDAP1L1 | 0.292386138 |
| USP39 | 0.361543459 |
| EDA | 0.632127193 |
| YARS | 0.635055499 |
| CXCL16 | 1.336666433 |
| SPON2 | 1.373765546 |
| C3 | 1.403288201 |
| SERPINE2 | 1.409576529 |
| STAU1 | 1.610300865 |
| FLNA | 1.736866543 |
| FGB | 1.854627201 |
| STAT3 | 1.923291708 |
| CTSB | 1.554385724 |
| HP | 2.384346611 |
| SERPINA3 | 4.324421317 |
| FGA | 1.984126984 |

The analysis refers to secreted proteins that were significantly changed in p53 dependent manner upon Nutlin-3a treatment [(sh-con treated/sh-con non-treated) <1.3 or >1.3, *t*-test P <0.05 and (sh-con treated/sh-p53 treated), or (fold change sh-con/fold change sh-p53) *t*-test P <0.05 n=3 independent experiments]

**Table S7:**

**Go biological annotations of p53 dependent secreted proteins altered upon CM from HCC-4006 cells**

| **GO biological annotations**  Miscellaneous signaling | | **Relevant proteins** |
| --- | --- | --- |
|  | Platelet Degranulation  Injury response | FGA, FGB, SERPINA3, FLNA |
|  | Hemostasis | FGA, FGB |
|  | Regulation of Fibrinolysis | FGA, FGB |
|  | Regulation of Plasminogen Activation | FGA, FGB |
|  | Positive Regulation of Peptide Hormone Secretion | FGA, FGB |
|  | Regulation of Endopeptidase Activity | SERPINE2, SERPINA3, C3 |
|  | Cellular Response to Leptin Stimulus | FGB, STAT3 |
|  | Signal Transducer Activity | FLNA, YARS, STAT3 |
|  | Response to Calcium Ion | FGA, FGB |
|  | Negative Regulation of Endothelial Cell Apoptotic Process | FGA, FGB |
|  | Toll-like Receptor Signaling Pathway | FGA, FGB, CTSB |
|  | Positive Regulation of Exocytosis | FGA, FGB |
|  | Neutrophil Degranulation  Immune response | SERPINA3, HP, CTSB, C3 |
|  | Inflammatory Response | SERPINA3, STAT3, C3 |
|  | Regulation of Immune System Process | SPON2, FGA, FGB, C3 |
|  | Response to Cytokine | CXCL16, STAT3 |
|  | Innate Immune Response  Cell motility | SPON2, FGA, FGB, C3 |
|  | Regulation of Cell Migration | SERPINE2, FLNA |
|  | Positive Regulation of Substrate Adhesion-dependent Cell Spreading | FGA, FGB, FLNA |
| ECM | Cell-matrix Adhesion | FGA, FGB, EDA |

**Table S8: Biological GO annotation of the p53-dependent secreted proteins following different treatment, related to Figure4**

| Biological GO pathway | | physiological conditions | Nutlin-3a | CM HCC-4006 |
| --- | --- | --- | --- | --- |
|  | development | v |  |  |
| Injury response | cytoskeleton | v |  |  |
|  | Blood coagulation | v | v |  |
|  | Hemostasis |  | v | v |
|  | fibrin | v | v | v |
|  | Plasminogen | v | v | v |
|  | Platelets | v | v | v |
|  | cellular metabolic | v | v |  |
|  | cholesterol | v | v |  |
|  | chylomicron | v | v |  |
|  | glycosaminogen  Metabolism | v | v |  |
|  | lipoprotein | v | v |  |
|  | retinoic acid | v | v |  |
|  | phosphatidylcholine | v | v |  |
|  | Keratin sulfate | v | v |  |
|  | lipids | v | v |  |
|  | glutamate | v |  |  |
|  | chondroitin | v |  |  |
|  | fatty acids | v |  |  |
|  | triglyceride | v |  |  |
|  | insulin |  | v |  |
|  | glycoside |  | v |  |
|  | glucocorticoid  ECM |  | v |  |
|  | ECM organization | v | v |  |
|  | ECM adhesion |  |  | v |
|  | stress fiber  Cell motility |  | v |  |
|  | cell adhesion |  | v | v |
|  | cell migration |  |  | v |
|  | leptin |  |  | v |
|  | transducer |  |  | v |
|  | calcium |  |  | v |
|  | apoptotic |  |  | v |
|  | exocytosis  Miscellaneous signaling |  |  | v |
|  | peptide hormone |  | v | v |
|  | endopeptidase | v | v |  |
|  | proteolysis | v | v |  |
|  | lysosome | v |  |  |
|  | autophagy | v |  |  |
|  | ERK1+2 | v |  |  |
|  | protein modification | v |  |  |
|  | fibroblast growth factor |  | v |  |
|  | tau protein |  | v |  |
|  | methionine |  | v |  |
|  | growth factor beta |  | v |  |
|  | WNT  Immune response |  | v |  |
|  | neutrophil |  | v | v |
|  | inflammation |  | v | v |
|  | immune process |  | v | v |
|  | cytokines in immune response |  | v | v |
|  | acute phase response |  | v | v |
|  | wound healing |  | v |  |
|  | Regulation of Complement Activation |  | v |  |
|  | innate response |  |  | v |

**Table S9- list of primers used for qRT-PCR, related to RNA isolation and quantitative real-time PCR (materials and methods).**

| **Gene name** | **Forward primer** | **Reverse primer** |
| --- | --- | --- |
| h-gapdh | ACCCACTCCTCCACCTTTGA | CTGTTGCTGTAGCCAAATTCGT |
| h-p21 | GGCAGACCAGCATGACAGATT | GCGGATTAGGGCTTCCTCTT |
| h-igfbp1 | TTGGGACGCCATCAGTACCTA | TTGGCTAAACTCTCTACGACTCT |
| h-serpine1 | GCACCACAGACGCGATCTT | ACCTCTGAAAAGTCCACTTGC |
| h-shbg | GCCCAGGACAAGAGCCTATC | CCTTAGGGTTGGTATCCCCATAA |
| h-igfbp2 | GACAATGGCGATGACCACTCA | CAGCTCCTTCATACCCGACTT |
| h-ctsb | GAGCTGGTCAACTATGTCAACA | GCTCATGTCCACGTTGTAGAAGT |
| h-ccl15 | TCCCAGGCCCAGTTCATAAAT | TGCTTTGTGAGATGTAGGAGGT |
| h-serpina3 | CCTGAAGGCCCCTGATAAGAA | GCTGGACTGATTGAGGGTGC |
| h-itm2b | TTGCCTCAGTCCTATCTGATTCA | TCTGCGTTGCAGTTTGTAAGT |
| h-npepps | GTGAGGCAGGCGACTAATCAG | GTTCCCGTACCTGTTTGCAG |
| h-hspg | CCAAATGCGCTGGACACATTC | CGGACACCTCTCGGAACTCT |
| h-matn3 | TCCAGGAAACCTTCTGTGCG | CACATCCGTGGGTGTTAAGAG |
| h-gpc1 | TGAAGCTGGTCTACTGTGCTC | CCCAGAACTTGTCGGTGATGA |
| h-saa4 | CTCTATGCTCGGGGAAACTATGA | TCAGCTTTCTCGTTGGACTTC |
| h-timp1 | AGAGTGTCTGCGGATACTTCC | CCAACAGTGTAGGTCTTGGTG |
| h-agrn | CCGCCAGGAGAATGTCTTCAA | TTTCGTAGGTGACTCCGTCGT |
| h-sema3f | AACACAACCGACTACCGAATC | GGCTGCCCAGTGTATAATGAG |
| h-gdf15 | ACCTGCACCTGCGTATCTCT | CGGACGAAGATTCTGCCAG |
| h-ctgf | CAGCATGGACGTTCGTCTG | AACCACGGTTTGGTCCTTGG |
| h-lolx4 | TCGTGGCTACCTTTCTGAAAC | GTGGCCCTCATACTTCACCTC |
| h-sulf2 | GGCAGGTTTCAGAGGGACC | GAAGGCGTTGATGAAGTGCG |
| h-serpine2 | TGGTGATGAGATACGGCGTAA | GTTAGCCACTGTCACAATGTCTT |
| h-lcn15 | GGACGGCTGTAACCAGGTG | CCAGGGTCGGGTAGAAGTC |
| h-pltp | CTCTCCACGTTCATCACCTCA | AATGCCAACAAGCTCGTCCA |
| h-dsc3 | GACCCTCGTGATCTTCAGTCG | TCACTTGACCGGATGAGGTCT |
| h-cel | TGGGTGACTCTGTGGACATCT | GCAGGCATCTCTTCTTGAAGTT |
| h-serpina6 | GTGAACATGAGTAACCATCACCG | CCTGGTGGATCTCAGTCTCAG |
| h-tigar | CTCTGACTGTTGTCCGGCAT | TGCATGGTCTGCTTTGTCCT |
| h-c4b | CCAGAAAGGCTACATGCGGA | CCCCCTGCATGCTCCTATG |
| h-p53 | CCCAAGCAATGGATGATTTGA | GGCATTCTGGGAGCTTCATCT |
| h-zeb1 | TTACACCTTTGCATACAGAACCC | TTTACGATTACACCCAGACTGC |
| h-zeb2 | CAAGAGGCGCAAACAAGCC | GGTTGGCAATACCGTCATCC |
| h-vimentin | GCAGGAGGCAGAAGAATGGTA | GCCTCAGAGAGGTCAGCAAACT |
| m-p21 | GGCCCGGAACATCTCAGG | AAATCTGTCAGGCTGGTCTGC |
| m-serpine1 | TTCAGCCCTTGCTTGCCTC | ACACTTTTACTCCGAAGTCGGT |
| m-rpl13a | AGCCTACCAGAAAGTTTGCTTAC | GCTTCTTCTTCCGATAGTGCATC |

**h-** Human gene

**m**- Mouse gene

**Table S10- list of primers used for PCR related to chromatin immunoprecipitation assay (materials and methods).**

| **Gene name** | **Forward primer** | **Reverse primer** |
| --- | --- | --- |
| h-p21 | CAAAATAGCCACCAGCCTCTTCT | AGCAGGCTGTGGCTCTGATT |
| h-igfbp1 | CTTCCAAAGCTCCTGCGTCTG | CTACCTGTGGTGATGTTGCCAC |
| h-serpine1 | GGGGAAGTCAGTCACAGAGC | TAGAGAAGGGGAGAGAGCCG |
| h-shbg | CTCCACCCTCAACCTCTCAA | AGGCATGAGAATCGCTTGAT |

**h-** Human gene

**Supplemental Experimental Procedures**

**Mass Spectrometry**

Sample preparation

10 ml of collected media were concentrated on a 3kDa NWCO Amicon filter (Millipore) and the retantate resuspended in 8M urea. 100 ug of total protein was reduced in 5mM 1,4-dithiothreitol (Sigma-Aldrich) for 1 hour at room temperature, followed by alkylation with 10mM iodoacetamide (Sigma-Aldrich) for 45 min in the dark. Trypsin (Promega) was added in a ratio 1:50 (trypsin:protein) and incubated overnight at 37^o^C. The digestion was stopped by acidifying the sample with 1% triflouroacetic acid (Sigma-Aldrich). The digested peptides were desalted on an Oasis column according to the manufacturer’s instructions (Waters) and vacuum-dried. The peptides were reconstituted in 3% acetonitrile (Sigma-Aldrich) and 0.1% formic acid (Sigma-Aldrich) prior to the LC-MS.

Liquid chromatography, mass spectrometry and data analysis

Liquid chromatography, mass spectrometry and data analysis were performed as previously described ^1^, following changes:

Peptides were eluted from the column into the mass spectrometer using the following gradient: 4% to 30% B in 150 min, 30% to 90% B in 5 min, maintained at 95% for 5 min and then back to initial conditions. For peptide identification, Mascot v2.5.1 was used against the uniprot human proteome. Data was normalized base on the total ion current. Protein abundance was obtained by summing the three most intense, unique peptides per protein. A Student’s t-Test, after logarithmic transformation, was used to identify significant differences across the biological replica. Fold changes were calculated based on the ratio of arithmetic means of the case versus control samples.

**References:**

1. Shalit T, Elinger D, Savidor A, Gabashvili A, Levin Y. MS1-based label-free proteomics using a quadrupole orbitrap mass spectrometer. *J Proteome Res* 2015, **14**(4)**:** 1979-1986.
